# A Case of Asphyxia from Drowning

**Published:** 1846-08

**Authors:** Henry Moreton

**Affiliations:** Assistant Physician to the Bellevue Hospital


*A Case of Asphyxia from Drowning.* By Henry Moreton, M. D., Assistant Physician to the Bellevue Hospital.—James Welch, æt. 46, born in Ireland, was observed on Tuesday, 2d of June, 1846, to throw himself into the East River, at the foot of 26th street, during a temporary aberration of mind. Assistance was procured as soon as possible, and he was rescued from the water, after having remained in it for the space of ten minutes.

He was immediately conveyed to the Bellevue Hospital, where I saw him soon after his admission. I found him completely asphyxiated—the whole surface of the body cold, the face livid, the veins of the neck very much distended, the pupils dilated and inactive, and a frothy mucus exuding from the mouth.

He was immediately wrapped in hot blankets, and insufflation attempted, but without success. Friction with mustard and vinegar was resorted to, one person being employed at each extremity. This treatment was continued for twenty minutes, when tr. cantharid. and tr. capsici. were substituted for the mustard and vinegar.

Warm brandy and ammonia were then poured into the mouth, but the function of deglutition, as well as that of circulation and respiration, was entirely suspended. A stimulating enema was then administered and retained.

In consideration of the congested state of the lungs and brain, I now consulted with Dr. N. Taylor, respecting the propriety of venesection, in order to relieve this surcharged condition of the nervous system, the result of which consultation was, that although depletion might restore the circulatory and respiratory movements, still, in the before-mentioned condition of the patient, general depletion was thought to be inadmissible; but as the same objections could not arise from the *local abstraction of blood*, cups were applied over the thoracic and epigastric regions—after which, slight pulsations were perceptible at the wrist, but no action of the respiratory muscles.

One hour had now elapsed since his admission, the cups were renewed and the friction continued for half an hour longer, without any change for the better; when, considering his case almost a hopeless one, I thought myself justified in resorting to a powerful derivative, and accordingly applied boiling water to his feet and legs, which to the surprise and gratification of Dr. Taylor and myself, as well as to the patients in the ward, acted promptly in restoring him, as the man immediately uttered an exclamation of pain, drew up his legs spasmodically, and in the course of two minutes was sensible, and reaction had thoroughly taken place.—*New York Medical and Surgical Reporter.—Boston Medical and Surgical Journal.*

